# A Small Community Model for the Transmission of Infectious Diseases: Comparison of School Closure as an Intervention in Individual-Based Models of an Influenza Pandemic

**DOI:** 10.1371/journal.pone.0004005

**Published:** 2008-12-23

**Authors:** George J. Milne, Joel K. Kelso, Heath A. Kelly, Simon T. Huband, Jodie McVernon

**Affiliations:** 1 School of Computer Science and Software Engineering, The University of Western Australia, Crawley, Western Australia, Australia; 2 Epidemiology Unit, Victorian Infectious Diseases Reference Laboratory, Carlton, Victoria, Australia; 3 School of Population Health, University of Melbourne, Carlton, Victoria, Australia; U.S. Naval Medical Research Center Detachment/Centers for Disease Control, United States of America

## Abstract

**Background:**

In the absence of other evidence, modelling has been used extensively to help policy makers plan for a potential future influenza pandemic.

**Method:**

We have constructed an individual based model of a small community in the developed world with detail down to exact household structure obtained from census collection datasets and precise simulation of household demographics, movement within the community and individual contact patterns. We modelled the spread of pandemic influenza in this community and the effect on daily and final attack rates of four social distancing measures: school closure, increased case isolation, workplace non-attendance and community contact reduction. We compared the modelled results of final attack rates in the absence of any interventions and the effect of school closure as a single intervention with other published individual based models of pandemic influenza in the developed world.

**Results:**

We showed that published individual based models estimate similar final attack rates over a range of values for R_0_ in a pandemic where no interventions have been implemented; that multiple social distancing measures applied early and continuously can be very effective in interrupting transmission of the pandemic virus for R_0_ values up to 2.5; and that different conclusions reached on the simulated benefit of school closure in published models appear to result from differences in assumptions about the timing and duration of school closure and flow-on effects on other social contacts resulting from school closure.

**Conclusion:**

Models of the spread and control of pandemic influenza have the potential to assist policy makers with decisions about which control strategies to adopt. However, attention needs to be given by policy makers to the assumptions underpinning both the models and the control strategies examined.

## Introduction

With continuing concern about the possibility of another influenza pandemic, many models have been developed to predict the course of the pandemic and the effect of potential intervention strategies. Approaches to modelling the spread of infectious respiratory diseases have included deterministic [1], [2], stochastic [3]–[5] and individual-based models [6]–[12]. These models have ranged in focus from the whole world [1], [3], [4], through large [8], [10] and small [7], [9] countries, to synthetic small communities [11]. However, while there are many individual-based models, no model constructed to date has focused on a precise replication of a small community, with detail down to individual schools, employers, and the exact make-up of households as extracted from census datasets.

We have developed a detailed spatio-temporal model of the Albany town and surrounding district. Albany, a relatively isolated community of approximately 30,000 people in the south of Western Australia, is a regional centre with one major hospital, one technical college, 22 schools and approximately 1200 employers. We believe that this modelled population provides us with a large enough experimental test-bed to capture the daily mobility of individuals as found in a developed nation. Using this model we examined the impact that social distancing measures might have in mitigating an influenza pandemic, given that social distancing measures can be implemented early in a pandemic by developed and developing countries alike.

We aimed to demonstrate the development of the model and its application to a human pandemic, with a pandemic virus spreading into the community from other parts of the state. We explored social distancing measures that included school (and child care) closure, reduced workplace attendance, reduced social and community contact, and increased home isolation of symptomatic individuals. These measures were examined for epidemics with basic reproduction numbers of 1.5, 2.0 and 2.5. The basic reproduction number (R_0_) is the average number of secondary cases that would be infected by a single infectious individual in a totally susceptible population, and is a measure of the transmissibility of an infectious disease. Since the R_0_ value of a new pandemic strain of influenza is unknown, we covered a range of R_0_ estimates for previous pandemics [7], [8], [13]. Furthermore we compared the baseline outputs of our model and the effects of a specific social distancing measure, school closure, with the results from other individual-based models.

## Methods

### Population model construction

We constructed a geographic and demographic model of Albany, Western Australia using a location-based connected spatial structure [3], [14]–[16]. Australian Bureau of Statistics Census Collection Districts (CCD) were the finest level of spatial detail used, with each CCD consisting of approximately 200 physically adjacent households. Each such area was populated with a number of households according to the 2001 census data [17]; the constituent households each being uniquely populated with individuals whose specific ages matched the demographics and household age-structure of each CCD.

The model was further populated with a set of schools and workplaces, referred to collectively as contact hubs. Data from the state government of Western Australia were used to obtain a comprehensive list of schools, childcare facilities, adult education institutions (provided by the Department of Education and Training; Frankland D, personal communication) and employers, including the location in which they were located and their nominal daytime population (provided by the Department of Planning and Infrastructure; Piscicelli A, personal communication). Each child was assigned to a school or childcare centre, presuming that children attended a school as close to their home location as possible, and ensuring that the known age structure of schools was maintained. Adult students and workers were assigned to adult education institutions and workplaces respectively, with this assignment being made with reference to census journey-to-work information survey data for Western Australia (provided by the Department of Planning and Infrastructure; Pradzynski J, personal communication).

### Application of the model to influenza infection

**Figure 1:**
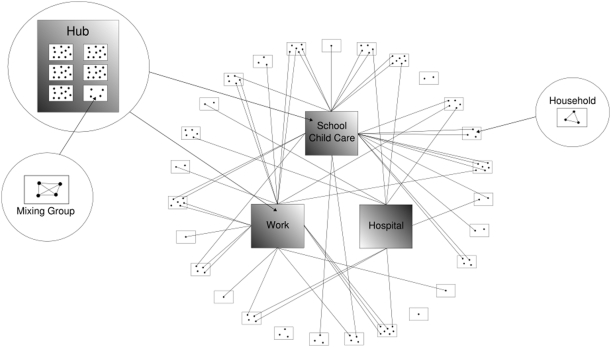
Idealised household and hub contact network.

Using this population model, we conducted stochastic, individual-based spatial simulations of an influenza epidemic, assuming that an average of one new infection per day was introduced into the population for the duration of the simulation. Each simulation proceeded in a sequence of 12 hour day/night cycles. During each cycle the nominal location of each individual was decided: either household or hub, taking into account the cycle type (i.e. day/night, weekend/weekday), the individual's infection status and whether an individual needed to stay at home to supervise a child. During each cycle, individuals occupying the same location were deemed to come into potential infective contact when infection transmission could occur. For larger hubs, including schools, we assumed it was unlikely that an individual would come into close contact with every other member of the hub during a cycle. These larger hubs were therefore divided into fixed mixing groups, with a maximum size of 10 individuals per group. In schools these mixing groups consisted of same-aged children where possible; in workplaces they were randomly assigned. The overlapping memberships of households and hubs formed a connected social network (see Figure 1). A study using mobile telephone location data has shown that human movement is dominated by a recurrent pattern with a 24-hour period [18], indicating that our assumption that individuals occupy two primary locations (a household and a hub) is a reasonable abstraction of human mobility in the developed world. Additional information about the population model construction and simulation algorithm can be found in Supplementary Information Text S1.

In addition to household and hub contact, individuals in the simulation also engaged in random or untraceable community contacts. This contact was assumed to be local in nature, with contacts between individuals from the same or nearby areas being relatively more likely than contact with individuals from distant patches.

When an infectious and susceptible individual came into contact during a simulation cycle, the probability that the infection was transmitted was calculated according to a *transmission function* (see below). For each contact event, an infection state (either to remain susceptible or to become infected) for the susceptible individual was randomly chosen via a Bernoulli trial [19]. The transmission probability *P_trans_* for a contact event is a function of the states of the infectious (*I_i_*) and susceptible (*I_s_*) individuals involved:
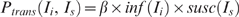
where *β* is the basic transmission coefficient, initially chosen to give an epidemic with a final attack rate consistent with seasonal influenza. To achieve simulations under a range of reproduction numbers, *β* was increased from the baseline value to achieve epidemics with target R_0_ values of 1.5, 2.0 and 2.5 (see Supplementary Info Table S1.1). R_0_ was derived by inserting an infectious individual randomly into a totally susceptible population, counting the number of resulting secondary infections, and averaging over 10,000 such trials. This is also the method used to derive R_0_ in [7]–[10]; additional information may be found in Supplementary Info Text S1.

The infectivity parameter *inf(I_i_)* was set to 1 for symptomatic individuals, and 0.5 for infectious but asymptomatic individuals. The susceptibility parameter *susc(I_s_)* is a function directly dependent on the susceptible person's age. It captures the age-varying susceptibility to transmission, due to both partial prior immunity and age-related differences in contact behaviour. To achieve a realistic age specific infection rate, the age-specific susceptibility parameters were calibrated against the serologic infection rates reported for H3N2 in 1977–1978 in Tecumseh, Michigan [20] (see Supplementary Information Table S1.2). We included in our sensitivity analyses an alternative calibration of age-specific susceptibilities that gave rise to a flat age-specific attack rate, similar to that of the 1968 pandemic [21] (see Supporting Information Text S2).

Influenza infection was modelled to last 6 days: 1 day latent, 1 day asymptomatic and infectious and 4 days infectious (either symptomatic or asymptomatic). We also assumed constant infectivity for the infectious period, which is a simplification of the presumed infectivity distribution found in studies of viral shedding [21], [22]. For baseline (no-intervention) epidemics with R_0_ values of 1.5, 2.0 and 2.5 these timing parameters gave serial intervals of 2.97, 2.87, 2.74 days respectively, which are consistent with previous estimates for pandemic and seasonal influenza [7], [13], [23].

Infected individuals were assumed to be immune to re-infection for the duration of the simulation. We also assumed that influenza symptoms developed 48 hours after infection with 20% of infections being asymptomatic in people aged 18 years or less and 32% being asymptomatic among older adults. These percentages were derived by summing the age-specific antibody titres determined in Table 5 of [24]. Symptomatic individuals were modelled to withdraw into the home with probability 50% for adults and 90% for children (ages 6–17).

We have used “illness attack rate” or “attack rate” to mean the proportion of the population who experience symptomatic infection, while “infection rate” refers to the proportion who were infected with symptomatic or asymptomatic infection.

### Application of the model to pandemic influenza

We assumed similar viral characteristics for pandemic influenza as we developed for seasonal influenza, as is suggested by existing data for H5N1 [25]. We further assumed a pandemic had been declared in South-East Asia, that the pandemic virus was already thought to be in Australia and that our modelled population was aware of the likely arrival of the pandemic strain. We therefore assumed a level of spontaneous social distancing in response to public health announcements and news reports. Such pandemic behaviour was assumed to contrast with that which occurs with seasonal influenza where overall mobility and contact patterns of asymptomatic individuals, and even some who are ill and symptomatic, remain unaltered. Baseline parameters were chosen to give rise to an epidemic with the following characteristics: an R_0_ of 1.5 giving a final illness attack rate of 34%, with 43% of infections occurring in households, 29% in schools and workplaces, and 26% from community contact. Based on seasonal influenza data [20] it has been estimated that 33%–37% of transmission occurs in the household [8]. Given that public knowledge of a current pandemic would induce spontaneous social distancing, we believe it reasonable to assume a lower level of community, school and workplace contact and a higher proportion of household transmission (43% rather than approximately 35%).

### Modelling interventions in a pandemic

We simulated four different non-pharmaceutical intervention measures as follows

#### School closure

We assumed that when schools were closed, students and teachers spent weekday daytime cycles at home rather than at school. This meant that no contact took place at that school hub, but that these individuals would contact any other individuals present in their household during the day cycle. We assumed that no *additional* community contact occurred (community contact was deemed to occur in all daytime cycles for active individuals, regardless of whether they were present at a hub or home). We also assumed that if school closure would result in a child being present in a household alone, one adult from the household stayed home (and did not make hub contacts). We assumed school closure applied to childcare facilities, all schools, and all adult educational institutions.

#### Increased case isolation

Our baseline assumption was that upon becoming symptomatic, there was a 50% chance that an adult, and 90% chance that a child, would withdraw to their household for the duration of their infection (infectivity and symptoms were deemed to cease at the same time). When the increased case isolation intervention was in effect, this increased house withdrawal to 90% for adults and 100% for children (ages 6–17). We assumed that withdrawn individuals made only household contacts while withdrawn.

#### Workplace non-attendance

When this measure was in effect, each person attending a (non-school) workplace hub had a 50% chance each day of staying home instead of attending the hub (the choice was made independently each day and applied only that day). Individuals staying at home made no hub contacts but did contact all other individuals also at home during the day cycle.

#### Community contact reduction

When this measure was in effect, it was assumed that individuals participating in community contact during a simulation cycle made 50% of the baseline number of effective contacts.

The degree of compliance to any intervention measure will obviously influence the effectiveness of the measure. For our simulations we have chosen parameters that, while severe, may be plausible in the context of a pandemic with significant mortality. Results of simulations examining the relationship between the degree of compliance and the derived effectiveness of each intervention are reported in Text S2.

### Comparison with other individual based studies

We reviewed the published results from five individual based simulation studies of the developing and developed world settings [7]–[11] and compared final infection rates from these studies and the effect of simulated school closure with the corresponding results from our own modelling. In some cases, symptomatic attack rates were reported; we converted these to infection rates using the asymptomatic infection proportion used by that study.

Additional information about this aspect of the study can be found in Supporting Information Text S1. All numerical model parameters are listed in Supporting Information Table S1.

## Results

### Pandemic characteristics with no interventions

We conducted baseline simulations (assuming no intervention measures) for epidemics with R_0_ values of 1.5, 2.0 and 2.5 As our simulations are stochastic in nature, the outcome of a simulated epidemic for a fixed set of model parameters varied depending, for example, on the choice of individuals who were “seeded” as infectious index cases; the outcome of possibly infectious contact; and the probabilistic contact behaviour of individuals. All results were averages of 40 independent simulation runs made with different random number seeding sequences. As we assumed a continuous influx of infectious cases from outside the simulation boundary at a rate of one per day, we achieve a sustained epidemic for every simulation. Final attack rates ranged from 33% to 65% corresponding to R_0_ values of 1.5 and 2.5, while peak daily attack rates ranged from 89 to 474 cases per 10,000 (Table 1).

**Table 1:** Simulated outcome of baseline (no-intervention) epidemics for three R_0_ values.

	R_0_ = 1.5		R_0_ = 2.0		R_0_ = 2.5	
	mean	(95% CI)	mean	(95% CI)	mean	(95% CI)
**Final infection rate (%)**	39.6	(±0.5)	66.7	(±0.2)	79.6	(±0.1)
**Final illness attack rate (%)**	33.2	(±0.4)	54.9	(±0.2)	64.8	(±0.1)
**Peak symptomatic population (%)**	5.3	(±0.17)	17.1	(±0.17)	28.3	(±0.17)
**Peak daily attack rate (cases per 10000)**	89	(±3.0)	279	(±3.6)	474	(±5.8)
**Peak attack day**	58	(±2.3)	37	(±1.0)	28	(±0.7)
**Serial interval (days)**	2.97	(±0.005)	2.87	(±0.004)	2.74	(±0.003)

Model parameters for the R_0_ = 1.5 epidemic were determined as described in the text. The fundamental transmission probability β was increased to give epidemics with measured R_0_ values of 1.5, 2.0 and 2.5. The statistics given for each baseline epidemic are means of 40 independent randomly seeded simulation runs (95% confidence intervals for the 40-run means are given in parentheses).

### Modelling social distancing interventions

We simulated the effects of four different non-pharmaceutical interventions: school closure, increased voluntary isolation of symptomatic individuals, workplace non-attendance and reduced community contact, with assumptions about each intervention as described above. We simulated the optimal application timing of the measures by assuming that the interventions were implemented prior to the introduction of the first infected case and continued indefinitely. In Table 2 we present figures capturing the cumulative and daily attack rates determined by simulated epidemics with (unmitigated) R_0_ values of 1.5, 2.0 and 2.5. We also conducted a series of simulations to determine the sensitivity of intervention measures to variation in key model parameters, including the degree of compliance, with results presented in the Supporting Information Text S2.

**Table 2:** Simulated final and peak daily attack rates for epidemics with non-pharmaceutical interventions.

Intervention scenario	R_0_ = 1.5		R_0_ = 2.0		R_0_ = 2.5	
	Final attack rate %	Peak daily attack rate (cases per 10000)	Final attack rate %	Peak daily attack rate (cases per 10000)	Final attack rate %	Peak daily attack rate (cases per 10000)
**Baseline**	33	89	55	279	65	474
**School Closure**	13	20	45	146	60	321
**Case Isolation**	6	9.0	30	78	49	221
**Workplace Nonattendance**	24	54	48	210	60	389
**Community Contact Reduction**	16	25	41	142.	55	291
**School Closure+Case Isolation**	3	4.0	8	12	30	67
**School Closure+Workplace Nonattendance**	6	10	34	80	54	25
**School Closure+Community Contact Reduction**	3	5.0	12	17	36	89
**All Measures**	2	3.0	2	4	3	5

Final attack rates and peak daily attack rates are given as percentages of the population, for epidemics with baseline R_0_ values of 1.5, 2.0 and 2.5. For each measure or combination of measures, results are given for optimal application (pre-emptive activation and indefinite duration). All results are means of 40 independent randomly seeded simulation runs.

For epidemics with an unmitigated R_0_ of 1.5, case isolation, school closure or community contact reduction made significant reductions in the final attack rate, reducing it from 33% to 6%, 13% and 16% respectively. School closure combined with any of the other interventions reduced the cumulative attack rate to below 10%, which may be deemed to be the threshold below which an epidemic does not occur. School closure combined with case isolation reduced the final attack rate to 8%. For epidemics with an R_0_ of 2.5, only the combination of all the modelled intervention measures appeared capable of controlling the epidemic, reducing the final attack rate from 65% to 3%. School closure combined with case isolation more than halved the final attack rate (to 30%). To achieve the large reductions in attack rates for R_0_ values of 2.0 and 2.5, combinations of interventions needed to operate for unfeasibly long periods of time (greater than 5 months).

The effect of interventions on peak daily attack rates followed a similar pattern to that of final attack rates, although the proportional reductions resulting from each intervention was larger than for final attack rates.

### Comparison with other studies

**Figure 2:**
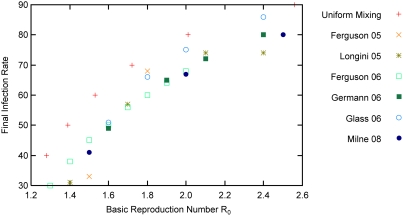
Simulated final infection rates plotted against basic reproduction number R_0_ for a number of epidemic models, assuming no intervention.

The characteristics of our baseline epidemics were consistent with other simulations based on stochastic individual-based models, specifically [7]–[11]. Figure 2 shows predicted final infection rates plotted against basic reproduction number R_0_ for five such models: orange (Ferguson et al 2005, Thailand) [7], light green (Ferguson et al 2006, United Kingdom) [8], brown (Longini et al 2005, Thailand) [9], dark green (Germann et al 2006, U.S.A.) [10], and light blue (Glass et al 2007, a synthetic 10,000 member community) [11]. Dark blue represents this study. The final infection rate predicted by a simple susceptible-infected-removed (SIR) differential equation model assuming uniform [26] is included for comparison (red). It should be noted that Figure 2 is similar to Figure 2 in the work of Roberts et al [2]. Both figures plot final infection rate against R_0_ for a number of individual-based models. Our figure adds additional data points for the Ferguson et al 2006 study [8] and includes two additional models: the Glass et al 2007 study [11] and the study described in this paper.

School closure was the most widely represented non-pharmaceutical intervention in comparable simulation studies. Three previous studies [8], [10], [11] simulated school closure as a sole intervention measure. Table 3 compares the effect of school closure for these studies and our own. The effect of school closure on final infection rates is shown, and for each study assumptions about the operation of school closure are summarised.

**Table 3:** Summary of Simulated Effectiveness of School Closure.

	Low R_0_		Higher R_0_		School Closure Assumptions
	R_0_	IR / IR with School Closure	R_0_	IR / IR with School Closure	
**Ferguson 2006**	1.7	54 / 48	2.0	68 / 64	Individual schools close for 3 weeks upon detection of case in school (schools can close multiple times); 10% workplace closure, additional household contact; increased household (50%) and community (25%) contact.
**Germann 2006**	1.6	48 / 1.5	1.9	65 / 44	Simultaneous and continuous school closure at 10,000 (29 or 24 days) cases plus 7 days; no additional contact.
**Glass 2006**	1.6	51 / 41 (4)	2.0	75 / 73 (50)	90% school closure compliance after 10 community cases. School closure infection rate given assuming additional contact and no additional contact in parenthesis.
**Milne 2008**	1.5	41 / 16	2.0	67 / 55	Schools close pre-emptively; additional household contact; adult required to supervise children in household.

Simulated effects of school closure on final infection rate for four individual-based influenza epidemic models. For each model results are given for moderate (R≤1.7) and severe (R≥1.9) epidemics. Each model's assumptions about the timing of the imposition of school closure and changes in mixing behaviour are summarised. Abbreviations: IR = Infection Rate.

Most models concluded that school closure would have only a modest effect when R_0_ was approximately 2.0. Ferguson et al [8] modelled school closure occurring for periods of 3 weeks triggered by the appearance of a case within the school, and found a 4% decrease in the infection rate (68% to 64%). The effect on the infection rate was more dramatic in the model of Germann et al, decreasing from 65% to 44%, but this model assumed that school closure caused no additional increase in interpersonal contact [10]. Early and continuous school closure in the model of Glass et al resulted in a decrease in the infection rate from 73% to 50% [11]. Our model, also assuming early and continuous school closure, resulted in a decrease in infection rate from 67% to 55%.

## Discussion

The scale of our model (approximately 30,000 individuals) is both small enough to allow the collection of detailed information and large enough to encompass the level at which public health planning and response might take place.

The model of Albany was constructed using a large number of parameters and is sensitive to the values assigned to them. We have utilised data from studies of past pandemics, from seasonal influenza epidemics and from related modelling work to set parameters but some parameters remain difficult to estimate. Sensitivity analyses, reported in Supporting Information Text S2, suggest that the model is most sensitive to assumptions regarding mixing group sizes in schools; the relative number of community contact, compared with household and hub contact; the proportion of asymptomatic infections; and the age-specific attack rate, specifically whether children are more susceptible to infection than adults.

### Potential impact of non-pharmaceutical interventions

We have examined the impact that non-pharmaceutical interventions may have on the course of a pandemic in a systematic manner, investigating a range of interventions in isolation and in combination. Our results suggest that the rapid activation of multiple non-pharmaceutical interventions may have a significant effect on slowing the rate of spread and reducing the final attack rate of an influenza pandemic in a small developed world community. These results hold for all reproduction numbers considered but are more effective for the lower numbers. Our model further suggests that for R_0_ = 2.5 a local epidemic may be prevented by the use of non-pharmaceutical measures alone, provided that activation of measures is rapid and sustained. While we acknowledge that the long-term enforcement of disruptive non-pharmaceutical interventions is not socially feasible, our results suggest that they have a key role to play, slowing the rate of growth of the pandemic until vaccination or antiviral drugs become available. Furthermore, many countries may not have access to a pandemic vaccine or to antiviral drugs, and if antiviral drugs are used a pandemic virus may rapidly become resistant [27]–[30], further highlighting the importance of non-pharmaceutical interventions.

### Comparison of the baseline pandemic and the effect of school closure simulations

Several previous studies [7]–[11] have used individual-based models to simulate the spread of influenza. By comparing the models (including our own), several conclusions can be drawn. Final infection rates, in the absence of any interventions, are similar in all models across a range of R_0_ values. This is true despite the fact that the models were constructed from a variety of data sources and took different approaches to building the simulated population and its implicit contact network. For epidemics with R_0_ values greater than 2.0, no single social distancing measure is effective in preventing a local epidemic.

For R_0_ in the range 1.5 to 2.0, different models make quite different predictions about the effectiveness of social distancing measures. The most commonly modelled and clearly comparable intervention is school closure. The effect of school closure as a sole intervention measure shows final infection rates ranging from 1.5% to 48% for R_0_ in the range 1.5–1.7. Given the similarity in modelled infection rates in the various individual based models in the absence of interventions, the difference in the effect of school closure appears to be related to the differences in assumptions about the contact behaviour of pupils during periods of school closure (an observation also made by Haber et al in [31]). For instance, the study by Germann et al [10] assumed that no additional contact occurred, and found school closure highly effective at R_0_ = 1.6, with a simulated final infection rate 1.5%. Our study (R_0_ = 1.5) assumed that additional household contact would occur and found that school closure would be moderately effective, reducing infection rate to 16%. The study by Ferguson et al [8] assumed that both household and community contact would increase and found that school closure for an R_0_ = 1.7 would only be marginally effective. The Glass et al study [11] supports the hypothesis that simulated school closure effectiveness is related to assumptions about mixing. For an R_0_ of 1.6, a scenario where pupils continued to contact their friends during school closure found such closure to be marginally effective (reducing infection rate to 41%), while a scenario in which this mixing did not occur resulted in school closure being highly effective (final infection rate 4%).

An alternative (or compounding) explanation for the variation in simulated effectiveness of school closure is the different assumptions regarding the timing of its introduction. The Ferguson et al [8] scenario, where individual schools close at the appearance of the first symptomatic case in the school, may result in closure occurring significantly later than the closure scenarios used in the other studies, all of which assume that all schools in a community close before cumulative symptomatic cases in a community reach approximately 10 cases per 10,000.

The stochastic modelling study described in [5] found that a comparison of attack rates between adults and children provided a good indication of the likely benefits of closing schools. In our sensitivity analysis (reported in Supporting Information Text S2) we found that an age-specific attack rate profile similar to the 1968 pandemic (where children had much the same attack rate as other age groups, rather than having a higher attack rate) did indeed reduce the effectiveness of school closure. However, we also found that altering assumptions about the size of school class mixing groups or the amount of community contact occurring also resulted in changes to the effectiveness of school closure, even when age-specific attack rates were the same.

This apparent lack of consensus highlights the sensitivity of individual-based models to the details of interpersonal contact and individual behavioural patterns, and suggests that obtaining reliable estimates of these parameters should be a priority. All the models suggest that decisions on school closure options, including when and for how long to close schools, will also have a major effect on the final infection rate in a community.

The results of our model, and of most of the other individual based models, are applicable to industrialised populations and may not be applicable to developing countries with lower population mobility and higher population densities. However, we have shown that published individual based models of developed world communities estimate similar final attack rates in a pandemic where no interventions have been implemented; that multiple social distancing measures applied early and continuously can be very effective in interrupting transmission of the pandemic virus for R_0_ values up to 2.5; and that different conclusions reached on the simulated benefit of school closure probably result from differences in assumptions about the timing and duration of school closure and flow-on effects on other social contacts resulting from school closure.

## Supporting Information

Text S1 Additional model details.
(0.07 MB DOC)

Text S2 Sensitivity analysis
(0.55 MB DOC)

Table S1 Baseline simulation parameters
(0.06 MB DOC)
