# Supplementary material for: A Small Community Model for the Transmission of Infectious Diseases: Comparison of School Closure as an Intervention in Individual-Based Models of an Influenza Pandemic
Source: PLoS One. 2008 Dec 23;3(12):e4005. doi: 10.1371/journal.pone.0004005 (PMC2602849; doi:10.1371/journal.pone.0004005)
Supplement: Text S1 — Additional model details. (0.07 MB DOC) [file pone.0004005.s001.doc]

**S****upporting Information Text S1 : Additional Method Details**

Additional model information

In addition to contact occurring in households and hubs (schools and workplaces), we also assumed that contact occurred between individuals within the wider community. This community contact was modelled as follows. During the daytime phase, every active individual made one or more (the number depending on the value of the community contact parameter) notional return trips, either within their home area or to a nearby area. Trip destinations areas were randomly chosen, weighted by population density and proximity to the traveller’s home area. For each of these community contact trips, the individual was randomly paired with another individual who also was making a trip to the same area, and a potentially infective contact occurred.

We assumed that if a susceptible person was exposed to multiple infectious persons during a simulation cycle, the probability of transmission from each was independent, with only one successful transmission being required for a susceptible person to become infected. Infected individuals were assumed to be immune to re-infection for the duration of the simulation.

Hospital model

The 120-bed Regional Hospital is explicitly included in our simulation model due to its role as the largest contact hub in Albany. Hospital admissions may occur from two sources; each symptomatic influenza case that occurs is deemed to have a 1% chance of resulting in hospitalisation. In addition, we assume that there is a “normal” flow of patients into and out of the hospital, modelled by a per-person, per-day probability of hospitalisation and discharge for the non-hospitalised and hospitalised population respectively. These parameters are adjusted so that the average hospital occupancy and length of patient stay matches actual hospital data obtained.

Contact within the hospital is deemed to take two forms. Firstly, we treat the hospital as a regular employment hub; this models mixing between hospital staff. In addition, the hospital is divided into “patient mixing groups” (which we call “rooms”), each containing a fixed number of beds (8). Each admitted patient is assigned to a room with a vacant bed; in addition, each room is associated with two hospital staff members (staff members can be associated with more than one room if there are less than twice as many staff as rooms). During every simulation phase (day and night), contact occurs between all pairs of patients in the same room, and between the staff associated with a room and each patient in the room.

R_0_ estimation and calculation

In our study we used R_0_, the basic reproduction number, as a measure of the severity of an epidemic, a standard approach in modelling studies. We derived R_0_ by seeding a single random infectious individual into a totally susceptible population, counting the number of secondary cases arising, repeating this process a large number of times (10,000 in our case), and taking the average.

This calculation produces a quantity that differs from the formal definition of R_0_. R_0_ is defined to be the average number of infections that would be caused by a *typical infected individual* in a totally susceptible population, whereas our method calculates the average number of infections that would be caused by a *randomly infected member of the population* in a totally susceptible population. This quantity is referred to as R_rand_ in [2].

The discrepancy between R_0_ and R_rand_ is small [2]. Note however that even if this is not the case, the results for our model can be interpreted without reference to R_0_ at all. We could equally have labelled our baseline epidemics as “33%, 55% and 65% unmitigated final attack rate epidemics” rather that “R_0_ 1.5, 2.0 and 2.5 epidemics”.

The majority of the models examined in this paper ([1,2,5,6]) approximate R_0_ by R_rand_. Adopting the same convention allows the comparison of model results even though the exact relationship between R_0_ and R_rand_ is not quantifiable.

Previous influenza pandemics of the twentieth century had R_0_ values estimated to be between 1.5 and 2.0 [1-4]. We included the higher R_0_ value of 2.5 in our study for several reasons. Firstly, some R_0_ values have been estimated from historical data where various intervention measures are known to have been active [1,2]. In contrast, we require an “unmitigated” R_0_ value for our baseline scenarios that assume no mandated intervention measures. Secondly, where estimates of unmitigated R_0_ have been made, estimates are based on a model that assumes uniform mixing [3,4]. As shown in Figure 2 of the main text, an individual-based model calibrated to a particular R_0_ value will significantly under-predict the final attack rate compared to a model that assumes uniform mixing. Thirdly, we wish to consider the unlikely but possible emergence of an extremely transmissible viral strain

Individual-based models comparison data sources

Data points for Figure 2 in the main paper were obtained from the following sources. In some cases, symptomatic attack rates are reported; these were converted to infection rates using the asymptomatic infection proportion used by that study.

| **Study** | **Reference** | **Data source** |
| --- | --- | --- |
| Ferguson et al 2005 | [1] | Caption of Figure 2d* |
| Longini et al 2005 | [2] | Figure 3a, 33% symptomatic proportion |
| Ferguson et al 2006 | [5] | Figure 1c, 50% symptomatic proportion |
| Germann et al 2006 | [6] | Table 2, 33% symptomatic proportion |
| Glass et al 2006 | [7] | Table 4 |
| Milne et al 2008 |  | Table 1 |

* R = 1.8 data point corrected to 68% from 50% in Supporting Information of [2].

The data points for the differential equation SIR model with uniform mixing are solutions to the equation:

ln ( 1 – *I* ) = - *R_0_ I*

where *I* is the final infection rate [8].

References

1. Ferguson NM, Cummings D, Cauchemez S, Fraser C, Riley S, et al. (2005) Strategies for containing an Emerging Influenza Pandemic in Southeast Asia. Nature 437: 209-214.
2. Ferguson N, Cummings D, Fraser C, Cajka J, Cooley P, et al. (2006) Strategies for mitigating an influenza pandemic. Nature 442: 448-452.
3. Caley P, Philp D, McCracken K (2007) Quantifying social distancing arising from pandemic. J R Soc Interface 5: 631-639.
4. Bootsma M, Ferguson N (2007) The effect of public health measures on the 1918 influenza pandemic in U.S. cities. Proc Nat Acad Science (USA) 104: 7588-7593.
5. Longini I, Nizam A, Shufu X, Ungchusak K, Hanshaoworakul W, et al. (2005) Containing pandemic influenza at the source. Science 309: 1083-1087.
6. Germann T, Kadau K, Longini I, Macken C (2006) Mitigation strategies for pandemic influenza in the United States. PNAS 103: 5935-5940.
7. Glass R, Glass L, Beyeler W, Min H (2007) Targeted social distancing design for pandemic influenza. Emerging Infectious Diseases 12: 1671-1681.
8. Anderson R, May R (1991) Infectious diseases of humans: dynamics and control. Oxford: Oxford University Press. 757 p.
