# Supplementary material for: A Small Community Model for the Transmission of Infectious Diseases: Comparison of School Closure as an Intervention in Individual-Based Models of an Influenza Pandemic
Source: PLoS One. 2008 Dec 23;3(12):e4005. doi: 10.1371/journal.pone.0004005 (PMC2602849; doi:10.1371/journal.pone.0004005)
Supplement: Text S2 — Sensitivity analysis (0.55 MB DOC) [file pone.0004005.s002.doc]

**Supporting Information Text S2 : Sensitivity Analysis**

Model parameter setting sensitivity

We examined the sensitivity of our simulation results to 8 key model parameters. We examined two alternative settings for each parameter, one significantly greater and the other significantly smaller than the value used for the standard baseline (standard baseline values are listed in Supplementary Information Table S1), giving sixteen additional sensitivity analysis scenarios. Table S2.1 describes each scenario.

| **Table S2.1** Sensitivity Analysis Scenarios | | |
| --- | --- | --- |
| **Parameter** | **Description** | **Values**  **(low, standard, high)** |
| *Behavioural Scenarios* | | |
| Background Contact | Number of daily contacts in the community by active individuals (each contact is equivalent to one simulation cycle of household or hub contact). | (2,4,8) |
| School Contact Group Size | Size of contact group in school hubs. | (5,10,20) |
| Maximum Work Group Size | Maximum size of contact groups in workplace hubs. | (5,10,20) |
| Illness Withdrawal | Probability that an adult will withdraw to household upon experiencing symptomatic infection.  Children always withdraw with probability 0.9, or 1.0 if case isolation is in effect. | (0.25,0.5,0.75) |
| *Biological Scenarios* | | |
| Infective Duration | Number of days during which infected individuals are infectious | (3,5,8) |
| Asymptomatic Infectiousness | Relative infectiousness of asymptomatic infected individuals. | (0.25,0.5,0.75) |
| Asymptomatic Proportion | Probability that an infected individual experiences asymptomatic infection. | (0.1,0.3,0.4) |
| Incubation Period | Delay in appearance of symptoms from infection (all infected cases become infectious after 24 hours). | (36 hrs, 48 hrs, 60 hrs) |
| Age-Specific Attack Rate | Attack rate of each age group. | “seasonal influenza”  (highest in children)  vs  “flat”  (same for all ages) |

For each scenario, the following procedure was carried out:

1. A set of age-specific susceptibility parameter values were determined so that the age-specific attack rates match, as closely as possible, that of the seasonal influenza profile recorded by the Tecumseh 1978-1979 study [1].[[1]](#endnote-2) These age-specific attack rates show proportionally higher attack rates in children and adolescents, and are similar to the 1957 influenza pandemic [2].[[2]](#footnote-2) This resulted in epidemics with R_0_ values of approximately 1.3 .
2. Each of these scenarios was simulated. Although constructed to have the same overall and age-specific attack rates, the epidemics do differ in other characteristics such as the proportion of transmission occurring in different types of location and the serial interval. Some characteristics of these baseline scenarios are given in table S2.2 (all epidemic outcomes reported here are averages of 40 independent randomly seeded simulation runs).
3. For each of the baseline scenario described in Table S1, a *β* parameter value (see the transmission function in the main text) was determined to ensure that the resulting epidemic had an R_0_ value of 1.5 .
4. For each of the R_0_ = 1.5 scenarios, the four different intervention measures examined in the main article were simulated, and the results compared to the corresponding no-intervention R_0_ = 1.5 baseline. It is assumed that the intervention measures are applied pre-emptively from the beginning of the simulation and continue indefinitely. Table S2.3 shows the sensitivity of each intervention measure to each scenario. For each intervention measure and each scenario, the reduction in final attack rate (as a percentage of the total population) is given. Where the scenario leads to a final attack rate reduction differing by greater than 7% from the reduction given by the standard baseline, the difference is also given (in red where the scenario results in a less effective intervention, and in green for a more effective intervention).

| **Table S2.2** Sensitivity Analysis Scenario Epidemic Characteristics (R_0_ ~ 1.3) | | | | | | |
| --- | --- | --- | --- | --- | --- | --- |
|  |  | **Final Infection Rate (%)** | **Serial Interval** | **% Home Transmissions** | **% Hub Transmissions** | **% Community Transmissions** |
| Standard baseline |  | 17.4 | 2.99 | 46 | 30 | 24 |
|  |  |  |  |  |  |  |
| Background Contact | **2** | 17.0 | 2.96 | 50 | 34 | 16 |
|  | **8** | 17.6 | 3.00 | 39 | 25 | 35 |
| School Contact Group Size | **5** | 16.9 | 3.02 | 49 | 22 | 28 |
|  | **20** | 17.1 | 2.92 | 40 | 40 | 20 |
| Maximum Work Group Size | **5** | 17.4 | 3.00 | 48 | 24 | 27 |
|  | **20** | 17.4 | 2.96 | 42 | 36 | 21 |
| Illness Withdrawal | **0.25** | 17.2 | 3.04 | 43 | 31 | 26 |
|  | **0.75** | 17.0 | 2.90 | 49 | 28 | 22 |
| Infective Duration | **3** | 17.3 | 2.54 | 45 | 30 | 24 |
|  | **8** | 17.7 | 4.72 | 47 | 28 | 24 |
| Asymptomatic Infectiousness | **0.25** | 16.3 | 3.20 | 49 | 27 | 23 |
|  | **0.75** | 17.2 | 2.84 | 43 | 31 | 25 |
| Asymptomatic Proportion | **0.1** | 17.1 | 3.02 | 45 | 30 | 24 |
|  | **0.4** | 17.4 | 3.06 | 39 | 35 | 26 |
| Symptom Latency | **36** | 17.7 | 2.96 | 48 | 27 | 24 |
|  | **60** | 17.6 | 2.99 | 43 | 32 | 25 |
| Age-Specific Attack Rate | **flat** | 18.0 | 3.04 | 38 | 29 | 32 |

Note that although the parameters for each scenario were chosen to give an overall infection rate as close as possible to 17.4%, the process of increasing the *β* parameter value does not uniformly increase infection (and thus attack) rates by the same amount. This is because each scenario has different degrees of repeated contact. In all cases the index case infects on average 1.5 other individuals; however, in scenarios where repeated contact is significant, infected individuals are more likely to encounter infected individuals (either the individual who infected them, or an individual who was also infected by their infector) than would be the case under a random mixing assumption, resulting in a reduced reproductive number.

The alternative assumptions represented by each scenario influence the effectiveness of the intervention measures in plausible ways. For each of the most significant deviations from the standard baseline assumptions we can postulate the following explanations:

Scenarios in which relatively less contact occurs in schools (where background contact is larger, school mixing group sizes are smaller, or where the age specific attack rate does not favour school-aged children) negatively impact and lessen the effectiveness of school closure. Conversely, if school mixing group sizes are larger, school closure is more effective.

Case isolation is significantly less effective if 40% of infections are asymptomatic (rather than 30%), as fewer individuals isolate themselves. Our standard baseline assumption is that 50% of symptomatic individuals voluntarily isolate themselves. If this figure is assumed to be 75% then case isolation is less effective.

The effectiveness of workplace non-attendance (the least effective of the studied interventions) is relatively robust to alternative parameter assumptions. The only significant deviation from the baseline assumptions is the assumption of a flat (equal for all ages) age-specific attack rate (rather than one with greater attack rates in younger age groups). In this case relatively more transmission occurs in workplace hubs, making workplace non-attendance more effective.

A flat age-specific attack rate results in relatively more community contact and transmission; community contact reduction is more effective under this assumption. Community contact reduction is less effective if 40% (rather than 30%) of cases are asymptomatic; this is more difficult to explain. A higher asymptomatic proportion slightly increases community transmission (as show in Table S2.2), increases hub transmission, and decreases household transmission. One hypothesis is that the pattern of epidemic spread has a significant spatial component (i.e. infection spreads preferentially from an area to adjacent areas via household and community transmission) that is effectively retarded by reduced community contact. With greater numbers of asymptomatic cases, infection could be more geographically dispersed through increased hub transmission and reduced case isolation (which restricts individuals to their household patch), and community contact reduction is therefore relatively less effective.

| **Table 2.3** Intervention Measures Sensitivity Analysis (R_0_ = 1.5) | | | | | | | | | | |
| --- | --- | --- | --- | --- | --- | --- | --- | --- | --- | --- |
|  |  | **Baseline Attack Rate** | **School Closure** | | **90% Case Isolation** | | **50% Workplace**  **Non-attendance** | | **50% Community Contact**  **Reduction** | |
|  |  |  | **Attack Rate (% of population)** | | | | | | | |
| Standard baseline |  | 34.1 | 23.5 |  | 29.8 |  | 8.1 |  | 19.2 |  |
|  |  |  |  |  |  |  |  |  |  |  |
| Background Contact | **2** | 28.7 | 25.4 |  | 24.8 |  | 9.2 |  | 12.8 |  |
|  | **8** | 36.0 | 12.4 | -11.1 | 32.0 |  | 6.4 |  | 25.5 |  |
| School Contact Group Size | **5** | 29.7 | 12.3 | -11.2 | 26.5 |  | 10.3 |  | 23.9 |  |
|  | **20** | 39.5 | 34.4 | +10.9 | 27.2 |  | 5.6 |  | 11.5 |  |
| Maximum Work Group Size | **5** | 34.8 | 24.4 |  | 29.5 |  | 4.0 |  | 20.5 |  |
|  | **20** | 33.4 | 20.9 |  | 29.1 |  | 11.9 |  | 15.6 |  |
| Illness Withdrawal | **0.25** | 33.4 | 20.9 |  | 30.5 |  | 9.6 |  | 20.9 |  |
|  | **0.75** | 33.0 | 26.3 |  | 22.6 | -7.2 | 6.0 |  | 16.5 |  |
| Infectious Duration | **3** | 35.4 | 24.7 |  | 27.8 |  | 7.8 |  | 18.4 |  |
|  | **8** | 26.8 | 20.2 |  | 24.2 |  | 8.4 |  | 19.4 |  |
| Asymptomatic Infectiousness | **0.25** | 30.6 | 22.4 |  | 28.1 |  | 7.4 |  | 20.2 |  |
|  | **0.75** | 36.8 | 23.0 |  | 25.6 |  | 7.9 |  | 18.2 |  |
| Asymptomatic Proportion | **0.1** | 36.7 | 25.7 |  | 31.9 |  | 8.4 |  | 19.4 |  |
|  | **0.4** | 29.1 | 18.3 |  | 12.2 | -17.6 | 5.1 |  | 11.7 | -7.5 |
| Symptom Latency | **36** | 25.9 | 19.0 |  | 23.5 |  | 9.8 |  | 20.3 |  |
|  | **60** | 36.6 | 26.1 |  | 24.2 |  | 7.3 |  | 18.2 |  |
| Age-Specific Attack Rate | **flat** | 33.9 | 13.8 | -9.8 | 31.0 |  | 15.2 | +7.1 | 27.7 | +8.5 |

Intervention measure compliance sensitivity

**
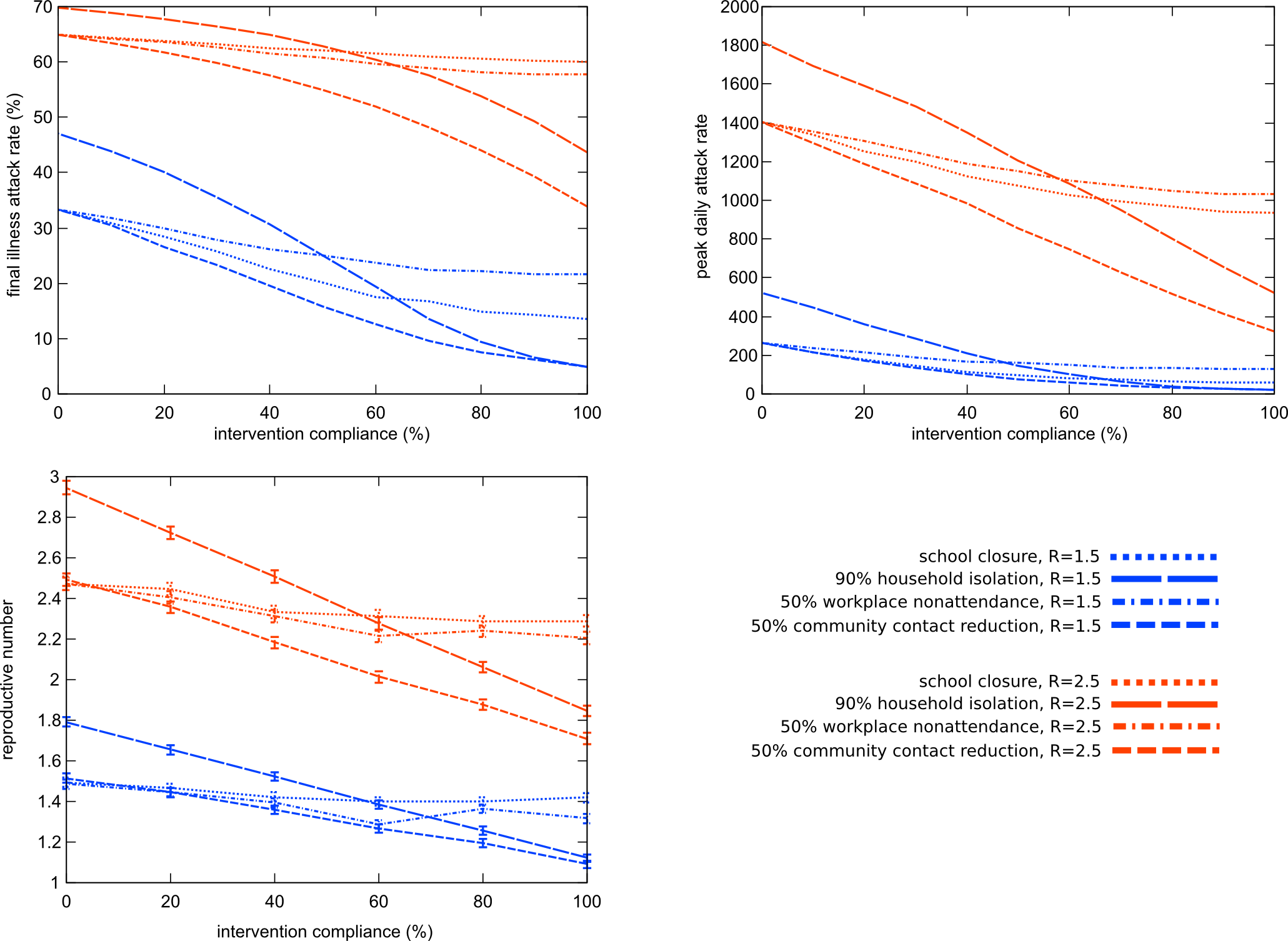
**

**Figure S2.1 Effect of degree of compliance to intervention measures on epidemic outcomes.**

The definition of “degree of compliance” for each intervention measure is given in the text below. Levels from 0-100% in 10% intervals were simulated.

(A) Final attack rate is plotted against the level of compliance for each of the four studied intervention measures, for R_0_=1.4 (blue) and R_0_=2.5 (red). (B) Similar to (A) but plotting peak daily attack rate. (C) Similar to (A), but plotting the reduction in R_0_ due to the operation of intervention measures (error bars show one standard deviation of the variance due to stochastic simulation).

Interventions are introduced pre-emptively and operate indefinitely.

Figure S2.1 shows the effect of the four intervention measures, assuming different degrees of compliance.

The definition of “degree of compliance” is particular to each intervention measure:

- For school closure compliance levels of less than 100%, it is assumed that schools remain open, but each day the probability that each student attends is equal to the compliance level. When at school, pupils make contact with other member of their usual mixing group that are also present. Our standard level of compliance for school closure is 100%.
- Our baseline (no intervention) assumption is that 50% (90% for children) of symptomatic individuals withdraw to their household for the period of their infection. Our standard level of compliance increases this to 90% for adults (and 100% for children), we examine levels from 0-100% for adults, assuming 100% for children.
- Our workplace non-attendance measure assumes that for each working day, the individual attends their workplace with a probability equal to the compliance level. The standard level of the workplace absenteeism measure is 50%.
- Our community contact reduction measure assumes that while the measure is in effect the quantity of community contact made by individuals is reduced by the compliance level. The standard level of compliance to community contact reduction is 50% (i.e. active individual make 50% fewer community contacts per day cycle).

The results are unsurprising, with higher compliance levels giving greater reductions in final attack rate and peak daily attack rates.

Most noticeable is that for the case isolation and community contact reduction measures, final and daily attack rates seem to decrease at least linearly with increasing compliance all the way up to 100% compliance, in contrast to the other measures where effectiveness levels off before approaching 100% compliance. This suggests that efforts to persuade individuals to isolate themselves are likely to be worthwhile, even if knowledge of the pandemic has already curtailed contact to some extent.

References

1. Monto A, Koopman J, Longini IMJ (1985) Tecumseh study of Illness. XIII. Influenza Infection and Disease, 1976-1981. American Journal of Epidemiology 121:881-822.

2. Davis L, Caldwell C, Lynch R, Bailey R, Chin T (1970) Hong Kong influenza: the epidemiologic features of a high school family study analyzed and compared with a similar study during the 1957 Asian influenza epidemic. American Journal of Epidemiology 92: 240-247.

1. [↑](#endnote-ref-2)
2. Except for the age-specific attack rate scenario; in this scenario age-specific susceptibilities were determined so that attack rates were the same in each age group (equal to the baseline illness attack rate of 17%). This age-specific attack rate profile is similar to that of the 1967 pandemic [2]. [↑](#footnote-ref-2)
