# Supplementary material for: A Small Community Model for the Transmission of Infectious Diseases: Comparison of School Closure as an Intervention in Individual-Based Models of an Influenza Pandemic
Source: PLoS One. 2008 Dec 23;3(12):e4005. doi: 10.1371/journal.pone.0004005 (PMC2602849; doi:10.1371/journal.pone.0004005)
Supplement: Table S1 — Baseline simulation parameters (0.06 MB DOC) [file pone.0004005.s003.doc]

| **Table S1** Baseline Simulation Parameters | | |
| --- | --- | --- |
| **parameter** | **meaning** | **value**(s) |
| *Transmission and Infection Characteristics* | | |
| *β* | Fundamental transmission probability. The basic probability of transmission for a cycle in which a susceptible individual and transmissible individual come into contact. | See table S1.1 |
| *susc(I_s_)* | Age-based susceptibility factor, a function of the susceptible individual (I_s_) age. | See table S1.2 |
| *inf(I_i_)* | Reduction in transmission probability for asymptomatic infected individual (I_I_), a function of their current symptom status. | 1.0, if I_i_ is symptomatic;  0.5, if I_i_ is asymptomatic |
| *Pr(asymptomatic)* | Probability that an individual experiences an asymptomatic infection. | 0.2, for ages 0-18;  0.32, otherwise |
| *Incubation Period* | Period of time between transmission and the point at which an infected individual becomes infectious. | 24 hours |
| *Symptom Latency* | Period of time between infection and onset of symptoms. | 48 hours |
| *Infection Duration* | Period of time from infection to end of symptoms and infectiousness. | 6 days |
| *Behavioural Characteristics* | | |
| *Pr(withdrawal)* | Probability that a symptomatic infected individual will withdraw to household upon appearance of symptoms. | 0.9 for ages 6-17;  0.5 otherwise. |
| *Withdrawal Period* | Period of time, from appearance of symptoms, that a withdrawing individual with remain at home | 4 days |
| *Community Contact* | Number of community contacts made per day by each active individual. | 4 |
| *Maximum Class Size* | Maximum size of mixing groups in schools, childcare centre and adult education institutions | 10 |
| *Maximum Workgroup Size* | Maximum size of workplace mixing groups. | 10 |
| *Seed Rate* | Average number of new infections imported into simulation per day. | 1.0 |
| *Seed Duration* | Number of days at beginning of simulation in which infections are seeded. | 365 |

| **Table S1.1** Fundamental per-contact transmission probability (β) | |
| --- | --- |
| **R_0_** | **β** |
| ~1.3 | 0.039 |
| 1.5 | 0.047 |
| 2.0 | 0.065 |
| 2.5 | 0.085 |

An initial value for β was derived by fitting age-based susceptibility factors to historical seasonal influenza age-specific attack rate data and normalising the adult susceptibility factor to 1.0 (see below). The measured R_0_ value for this parameter set was approximately 1.3 . To obtain epidemics with (unmitigated) R_0_ values of 1.5, 2.0 and 2.5, β was then raised to achieve the target R_0_ .

| **Table S1.2**  Age-specific attack rates and relative susceptibility parameters. | | | | |
| --- | --- | --- | --- | --- |
|  | **Seasonal influenza age specificity** | | **“flat” age specificity** | |
| **Age Category** | **Age-specific serological attack rate** | **Susceptibility factor** | **Age-specific serological attack rate** | **Susceptibility factor** |
| 0-5 | 30.0 | 1.50 | 17.4 | 0.82 |
| 6-12 | 29.2 | 1.14 | 17.4 | 0.71 |
| 13-17 | 28.0 | 1.63 | 17.4 | 0.88 |
| 18-24 | 21.6 | 1.76 | 17.4 | 0.97 |
| 25-44 | 17.0 | 1.00 | 17.4 | 1.00 |
| 45-64 | 7.0 | 0.52 | 17.4 | 1.22 |
| 65+ | 1.0 | 1.44 | 17.4 | 2.13 |

Susceptibility factors were found by optimising susceptibility factors until a target age-specific attack rate vector was matched within a desired tolerance (the root mean square deviation from target age-specific attack rate vector was within 0.5% of the total population with 95% confidence). Susceptibility factors have been scaled so that adult susceptibility = 1.0 .

The “seasonal influenza” age-specific attack rates are those reported in the Tecumseh study[[1]](#endnote-2) for the H3N2 influenza type in 1977-78. The “flat” age-specific attack rate assumes the same whole-of-population attack rate as the seasonal influenza (17.4), but assumes that the attack rate is the same in each age category.

1. Monto A, Koopman J, Longini IMJ (1985) Tecumseh study of Illness. XIII. Influenza Infection and Disease, 1976-1981. American Journal of Epidemiology 121:881-822. [↑](#endnote-ref-2)
